# 10-year mortality among first-time mothers involved in family court care proceedings in England: cohort study using linked administrative hospital, mortality and family court records

**DOI:** 10.1136/jech-2026-225930

**Published:** 2026-06-23

**Authors:** Georgina Ireland, Bianca De Stavola, Ruth Gilbert, Matthew A Jay

**Affiliations:** 1 Population, Policy & Practice Research & Teaching Department, GOS Institute of Child Health, UCL, London, UK; 2 Family Law & Health Research Group, GOS Institute of Child Health, UCL, London, UK

**Keywords:** RECORD LINKAGE, DEATH, MATERNAL HEALTH, CHILD

## Abstract

**Background:**

Family court care proceedings are instigated to remove children at risk of harm from parental care. Limited information is available on the health of mothers involved in care proceedings. We assessed maternal mortality and causes of death within 10 years of first birth, comparing first-time mothers with and without care proceedings.

**Methods:**

Using linked, administrative hospital and family court data, we followed a whole-population cohort of first-time mothers delivering between 2007 and 2017 up to 10 years. We calculated mortality rates comparing mothers with and without care proceedings. We examined proportions of deaths potentially preventable (suicide, homicide, drugs/alcohol or injury) and identified factors associated with death after care proceedings.

**Results:**

Of 2 775 835 first-time mothers contributing 21 856 503 person-years of observation, 28 405 (1.0%) had proceedings. Following proceedings, 314 (1.1%) died, compared with 5103 (0.2%) among mothers without care proceedings (age-standardised mortality ratio 21.0, 95% CI 14.3 to 27.7). Mortality ratios were lowest among first-time mothers aged <20 years (4.5, 95% CI 3.5 to 5.9) and highest for those aged 30–34 years (28.3, 95% CI 21.3 to 37.5). Among mothers who died after proceedings, 73% of deaths were potentially preventable compared with 28% among mothers without proceedings. Factors associated with death were older maternal age at proceedings, health conditions and court orders related to child removal.

**Conclusion:**

First-time mothers with care proceedings had 21 times the risk of dying within 10 years than similar-aged mothers. Healthcare, social care and family courts must address the extreme health vulnerability of mothers before, during and after proceedings.

**WHAT IS ALREADY KNOWN ON THIS TOPIC**

**WHAT THIS STUDY ADDS**

- This study offers the first analysis of premature mortality risk within 10 years of a first live birth for women involved in care proceedings compared with the general population. We found that the age-adjusted risk of death in periods following care proceedings was 21 times higher compared with periods without care proceedings.

**HOW THIS STUDY MIGHT AFFECT RESEARCH, PRACTICE OR POLICY**

- Better upstream prevention (by maternity, early childhood and family services) and family court reform are required to improve health, prevent the need for care proceedings and, where they are necessary, ensure that proceedings and post-proceedings support meet the needs of mothers.

## Background

 To prevent harm, child protection (part of children’s social care in England), universal healthcare and education services have duties to identify and refer to social care children who are experiencing abuse, domestic violence and neglect.^1^ Parents in England may lose child custody in such circumstances either through agreement with child protection services or by a mandatory order made by the family court in care proceedings (also known as public law family proceedings). Such court action represents one of the most serious interventions in family life available to the state and results in removal of a child in most instances.^2^

Maternal mortality is a benchmark for healthcare services and maternal health globally, yet longer-term survival following childbirth^3^ or following care proceedings is rarely studied. Increased mortality among mothers experiencing child custody loss through action by child protective services and/or family court in south London (England),^4^ Manitoba (Canada)^5 6^ and Sweden^7^ has been observed. In these studies, hazard ratios (HRs) of death ranged from 2 to 5 comparing those with and without custody loss. Mechanisms that explain this higher risk are likely complex, involving pre-existing poor mental and physical health,^4–9^ experience of domestic abuse leading to the proceedings as well as a history of abuse and neglect from childhood, the trauma of child removal,^10^ fear of accessing, or inability to access, public services, including healthcare,^11^ and systemic barriers and lack of support and protection before, during and after proceedings.^10 12^

Prioritising public health interventions requires understanding excess mortality among mothers experiencing care proceedings. Previous studies, however, underestimate differences with the general population by using similarly deprived comparison groups (such as siblings without child removal) and controlling for confounders through matching or statistical adjustment. A descriptive study is needed which uses a general population comparison group for all of England.^13^ We therefore aimed to compare 10-year mortality for first-time mothers during the period following care proceedings with similar age mothers who did not experience care proceedings in England.

Our objectives were to: (1) estimate the maternal mortality rate from first live birth according to whether the mother had experienced care proceedings or not; (2) describe the proportions of potentially preventable deaths (related to suicide, homicide, drug or alcohol use, or accidental injury) and those related to medical or other causes; and (3) identify which factors, including maternal social and health characteristics and proceedings outcome, were associated with mortality after care proceedings.

Findings could inform possible interventions in the early childhood years to improve the health of vulnerable mothers involved in care proceedings. We reasoned that care proceedings indicate health and social vulnerability, regardless of the legal outcome, and that courts, social care (who bring the case to court) and healthcare services have an opportunity and duty to intervene to mitigate risks to the mother.

## Methods

### Population and data sources

We included a cohort of all mothers with a first live birth while aged 15–39 years between 2007 and 2017. We focused on first-time mothers as early preventive interventions for mothers for their first child can benefit planning for, and outcomes of, subsequent children. The cohort was derived from a cohort of all women (15–50 years) giving birth in National Health Service (NHS) maternity units, available in Hospital Episode Statistics (HES) inpatient hospital records, detailed elsewhere.^14^ This cohort contained 92.9% of all births registered in England by the Office for National Statistics (ONS) between 1998 and 2021. Mothers were followed for up to 10 years following first live birth recorded in a hospital admission. Hospital admission records contain mandatory diagnostic data using the International Statistical Classification of Diseases and Related Health Problems, 10th revision (ICD-10) for each hospital episode, with one primary diagnosis (the primary cause of the admission) and up to 19 secondary diagnoses. Death registration records include cause of death (ICD-10; underlying cause of death and up to 15 contributory causes).

Administrative data for mothers (15–50 years at start of court case) involved in family court care proceedings in England between April 2007 and March 2022 were linked to the delivery cohort. Data were provided by the Children and Family Court Advisory and Support Service (Cafcass).^15^ This dataset included, for all family court care proceedings in England, de-identified information on hearing dates, number and ages of children involved, maternal demographic information, and orders made at the end of the proceedings. NHS England linked Cafcass identifiers to the Personal Demographic Service (a record of all patients NHS registered in England) via a deterministic algorithm with name, date of birth, sex (all female) and address (three most recent addresses). From there, data were linked to HES and a study-specific bridging file was supplied to us. This resulted in 83% of mothers involved in care proceedings in England linking to a mother in the delivery cohort. Further details are reported elsewhere.^14^

### Outcomes

For objective 1 (mortality rates), the outcome was mortality within 10 years of first live birth. Mothers were recorded as having died based on a death recorded in ONS registration records or an inpatient admission whose discharge method or destination was coded as death, with no record of later activity in hospital data. In the case of objective 2 (causes of death), we used cause of death data available in ONS death registration data (underlying cause and up to 15 contributory causes). Cause was not available where death was identified solely on the basis of hospital discharge (0.9% (n=49) of deaths). Additionally, cause of death was missing where there was a pending coroner’s investigation to determine registrable cause for deaths (affecting deaths in 2020 and not completed by 2022, estimated to be fewer than 0.2% of deaths). For objective 3 (factors associated with death following care proceedings), the outcome was mortality within 10 years of the start of care proceedings.

### Characteristics

We first described the characteristics of mothers involved and not involved in care proceedings: age at first delivery, racial-ethnic group, Index of Multiple Deprivation fifths,^16^ financial year of delivery, and the presence of health problems in the 3 years before birth and in the 3 years before the start of care proceedings. Health conditions were identified using previously developed ICD-10 code lists in hospital data. We examined the following overlapping phenotypes: long-term health conditions,^17^ physical or sensory disability,^18^ intellectual disability,^19^ mental health or behavioural conditions^4^ and adversity-related admissions (ie, emergency admissions related to violence, self-harm or drug/alcohol use).^20^ For objective 3, we classified the final legal order as: (1) case discharge or order of no order; (2) family assessment order or supervision order; (3) residence order, special guardianship order or child arrangements order; (4) care order or secure accommodation order; or (5) placement order. Discharges, orders of no order, family assessment orders and supervision orders usually mean that the child remains at home. In the case of the other orders, the child is likely to have been removed from maternal care.

### Statistical analysis

For objective 1 (comparing rates of death within 10 years of first live birth), we estimated 10-year mortality rates by care proceeding involvement. We followed all mothers from their first live birth until the first of 10 years, 51st birthday, 31 December 2020 or death. To address immortal time bias induced by the fact that mothers in the care proceedings group could not have died before experiencing proceedings, person-time before the start of care proceedings was categorised as time not exposed and included in the no-proceedings group. Crude rates by care proceedings (not—or not yet—experienced vs experienced) were calculated as the number of deaths over person-years, overall and in 5-year age bands. To account for different age structures in the two groups, we also calculated directly age-standardised mortality rates using the age structure of the whole study cohort as the reference cohort. We calculated age-specific and age-standardised mortality rate ratios. Standardisation was for the whole population of first-time mothers.

For objective 2 (causes of death), we described the proportions of deaths within 10 years of first birth that were potentially preventable—ie, related to suicide, homicide, drugs/alcohol or accidental injury—or related to medical, other or unknown causes by involvement in care proceedings and age group. Classification was based on ICD-10 codes (available on GitHub, see below). We compared mothers involved and never involved in care proceedings and we used two age groups (<25 or 25+ years at birth) due to small numbers in the finer 5-year age groups.

To address objective 3 (factors at proceedings associated with mortality), the cumulative mortality rate among the care proceedings group was estimated using the Kaplan-Meier method, starting from the start of care proceedings. We report this overall and stratified by age, deprivation at the start of proceedings, health conditions in the 3 years before case start, and final legal order at the end of proceedings. We do not present Kaplan-Meier curves due to low cell counts and statistical disclosure control. As our aim was to describe the factors associated with mortality in real world populations and not elucidate causal mechanisms, we provide unadjusted results.^13^

Code for creating the deliveries cohort and the cohort used in this study and all data processing and analysis code was written in R 4.4.1. Data were stored and analysed on the UCL Data Safe Haven, a secure research environment for handling sensitive data. This code and machine-readable copies of the ICD-10 code lists are available on GitHub at https://github.com/UCL-CHIG/deliveries-cohort for creation of the deliveries cohort and https://github.com/UCL-CHIG/cafcass-mortality for further processing and analyses for this study. Small cell counts (<10) and estimates based on these were suppressed as statistical disclosure control.

## Results

We included 2 775 835 women with a first live birth recorded in hospital between April 2007 and December 2017 in England. Of these, 28 405 (1.0%) experienced care proceedings within 10 years of their first live birth (median (IQR) 2.9 (1.0–5.6) years). Mothers involved in care proceedings were younger, more deprived and had more health problems recorded in the 3 years before birth than other mothers (table 1).

**Table 1:** Characteristics of mothers at first birth between April 2007 and December 2017 and number and percent who died within 10 years, by involvement in care proceedings or not within 10 years

		Experienced care proceedings		Did not experience care proceedings	
		Mothers n (%)	Deaths n (%)	Mothers n (%)	Deaths n (%)
Total		28 405	314	2 747 430	5103
Age at delivery (years)	15–19	13 954 (49.1%)	70 (22.3%)	244 032 (8.9%)	453 (8.9%)
	20–24	8913 (31.4%)	91 (29.0%)	619 587 (22.6%)	930 (18.2%)
	25–29	3100 (10.9%)	70 (22.3%)	818 982 (29.8%)	1282 (25.1%)
	30–34	1603 (5.6%)	50 (15.9%)	746 559 (27.2%)	1416 (27.7%)
	35–39	835 (2.9%)	33 (10.5%)	318 270 (11.6%)	1022 (20.0%)
Ethnicity	White	24 988 (88.0%)	292 (93.0%)	2 087 042 (76.0%)	4135 (81.0%)
	Black	1084 (3.8%)	22 (7.0%)*	114 967 (4.2%)	285 (5.6%)
	Mixed	634 (2.2%)	•	44 125 (1.6%)	95 (1.9%)
	Asian	821 (2.9%)	•	263 878 (9.6%)	345 (6.8%)
	Other	509 (1.8%)	•	116 436 (4.2%)	154 (3.0%)
	Unknown	369 (1.3%)	•	120 982 (4.4%)	89 (1.7%)
IMD fifth	1 – most deprived	13 386 (47.1%)	157 (50.0%)	681 359 (24.8%)	1397 (27.4%)
	2	7069 (24.9%)	64 (20.4%)	631 633 (23.0%)	1156 (22.7%)
	3	4010 (14.1%)	47 (15.0%)	537 124 (19.6%)	973 (19.1%)
	4	2356 (8.3%)	30 (9.6%)	461 891 (16.8%)	793 (15.5%)
	5 – least deprived	1470 (5.2%)	16 (5.1%)	427 705 (15.6%)	764 (15.0%)
	Not reported	114 (0.4%)	•	7718 (0.3%)	20 (0.4%)
Delivery financial year starting	2007–2009	9795 (34.5%)	135 (43.0%)	813 849 (29.6%)	2120 (41.5%)
	2010–2012	8897 (31.3%)	113 (36.0%)	774 214 (28.2%)	1767 (34.6%)
	2013–2015	6585 (23.2%)	53 (16.9%)	697 692 (25.4%)	891 (17.5%)
	2016–2017	3128 (11.0%)	13 (4.1%)	461 675 (16.8%)	325 (6.4%)
Health problems in 3 years before delivery	Any†	10 863 (38.2%)	184 (58.6%)	355 546 (12.9%)	1396 (27.4%)
	Long-term	4222 (14.9%)	80 (25.5%)	219 100 (8.0%)	829 (16.2%)
	Disability	1267 (4.5%)	29 (9.2%)	40 022 (1.5%)	267 (5.2%)
	Intellectual disability	924 (3.3%)	11 (3.5%)	3610 (0.1%)	20 (0.4%)
	Mental health/behavioural	6597 (23.2%)	153 (48.7%)	117 206 (4.3%)	524 (10.3%)
	ARA (any)	4394 (15.5%)	107 (34.1%)	42 041 (1.5%)	310 (6.1%)
	ARA (drugs/alcohol)‡	3833 (13.5%)	100 (31.8%)	36 836 (1.3%)	280 (5.5%)
	ARA (self-harm)‡	3095 (10.9%)	66 (21.0%)	28 796 (1.0%)	182 (3.6%)
	ARA (violence)‡	488 (1.7%)	<10 (<3.2%)§	3589 (0.1%)	23 (0.5%)
Health problems in 3 years before case start	Any†	12 038 (42.4%)	213 (67.8%)	•	•
	Long-term	4695 (16.5%)	96 (30.6%)	•	•
	Disability	1451 (5.1%)	31 (9.9%)	•	•
	Intellectual disability	934 (3.3%)	13 (4.1%)	•	•
	Mental health/behavioural	8803 (31.0%)	186 (59.2%)	•	•
	ARA (any)	3803 (13.4%)	110 (35.0%)	•	•
	ARA (drugs/alcohol)‡	1032 (3.6%)	43 (13.7%)	•	•
	ARA (self-harm)‡	359 (1.3%)	<10 (<3.2%)§	•	•
	ARA (violence)‡	103 (0.4%)	<10 (<3.2%)§	•	•
Final legal order	Discharge/ONO	1953 (6.9%)	14 (4.5%)	•	•
	FAO/SO	3358 (11.8%)	19 (6.1%)	•	•
	RO/SGO/CAO	9022 (31.8%)	102 (32.5%)	•	•
	CO/SAO	5908 (20.8%)	68 (21.7%)	•	•
	PO	5603 (19.7%)	78 (24.8%)	•	•
	Not reported	2561 (9.0%)	33 (10.5%)	•	•

* Includes all ethnic groups other than White due to small numbers.

† Any health problem includes long-term conditions, disability, intellectual disability, mental health/behavioural and any ARA. All are potentially overlapping.

‡ Potentially overlapping sub-groups of ARA.

§ Suppressed due to small cell count.

ARA, adversity-related admission; CAO, child arrangements order; CO, care order; FAO, family assessment order; IMD, Index of Multiple Deprivation; ONO, order of no order; PO, placement order; RO, residence order; SAO, secure accommodation order; SGO, special guardianship order; SO, supervision order.

### Objective 1: mortality from first birth

There were 314 (1.1%) deaths in 28 405 mothers involved in care proceedings and 5103 (0.2%) deaths in 2 747 430 mothers without care proceedings (median (IQR) follow-up time of those in care proceedings 9.5 (6.8–10.0) years vs 8.8 (6.0–10.0) of those without proceedings) (table 2).

**Table 2:** Crude mortality rates, crude mortality rate ratios and age-standardised rate ratio for mothers in periods after experiencing care proceedings compared with periods without or yet without care proceedings followed for up to 10 years after first live birth, overall and by maternal age at delivery

Age at birth (years)	Periods after care proceedings*			Periods without care proceedings*			
	Person-years	Deaths	Mortality rate (per 1000 person-years) (95% CI)	Person-years	Deaths	Mortality rate (per 1000 person-years) (95% CI)	Mortality rate ratio (95% CI)
15–19	71 171	70	0.98 (0.75 to 1.21)	2 093 795	453	0.22 (0.20 to 0.24)	4.5 (3.5 to 5.9)
20–24	40 114	91	2.27 (1.80 to 2.73)	5 021 986	930	0.19 (0.17 to 0.20)	12.3 (9.9 to 15.2)
25–29	13 841	70	5.06 (3.87 to 6.24)	6 429 070	1282	0.20 (0.19 to 0.21)	25.4 (19.9 to 32.3)
30–34	7254	50	6.89 (4.98 to 8.80)	5 804 654	1416	0.24 (0.23 to 0.26)	28.3 (21.3 to 37.5)
35–39	4063	33	8.12 (5.35 to 10.89)	2 506 998	1022	0.41 (0.38 to 0.43)	19.9 (14.1 to 28.2)
All mothers (crude)	136 443	314	2.30 (2.05 to 2.56)	21 856 503	5103	0.23 (0.23 to 0.24)	9.9 (8.8 to 11.1)
All mothers (age-standardised)			4.89 (3.37 to 6.42)			0.23 (0.22 to 0.25)	21.0 (14.3 to 27.7)

* To avoid introducing immortal time bias, the follow-up periods before care proceedings are included in the total of person-years without care proceedings.

The unadjusted rate of death within 10 years of birth for the periods after proceedings was 2.30/1000 person-years, compared with 0.23/1000 person-years for the periods without (table 2). The mortality rate after involvement in care proceedings increased with maternal age at first birth, from 0.98/1000 person-years in mothers 15–19 years of age to 8.12/1000 person-years for mothers aged 35–39 years (table 2). By comparison, the mortality rate where mothers had not (or not yet) experienced care proceedings was similar across all ages at around 0.20/1000 person-years, except for those aged 35–39 years at birth, where it was 0.41/1000 person-years.

The crude mortality rate ratio comparing periods of time following care proceedings with those without was 9.9 (table 2). The relationship between increasing age and mortality rate ratio was non-linear, being smallest in the 15–19 years age group (4.6) and highest in the 30–34 years group (28.3). These differences reflect an increase in the absolute rates with age among mothers with care proceedings that was not seen among mothers without proceedings. The age-standardised mortality ratio was 21.0, meaning that if both groups had the same age structure, the rate of death in periods following care proceedings would be 21 times higher than in periods without.

### Objective 2: causes of death

Among mothers with care proceedings, 229 (73%) of deaths were potentially preventable (related to suicide, homicide, drugs/alcohol or accidental injury) compared with 1405 (28%) among mothers without proceedings (figure 1; online supplemental table 1). There was no association with age among the care proceedings group whereas among those without care proceedings, the proportion of potentially preventable deaths was higher among the younger mothers (aged <25 years at first live birth; figure 1; online supplemental table 1).

**Figure 1:**
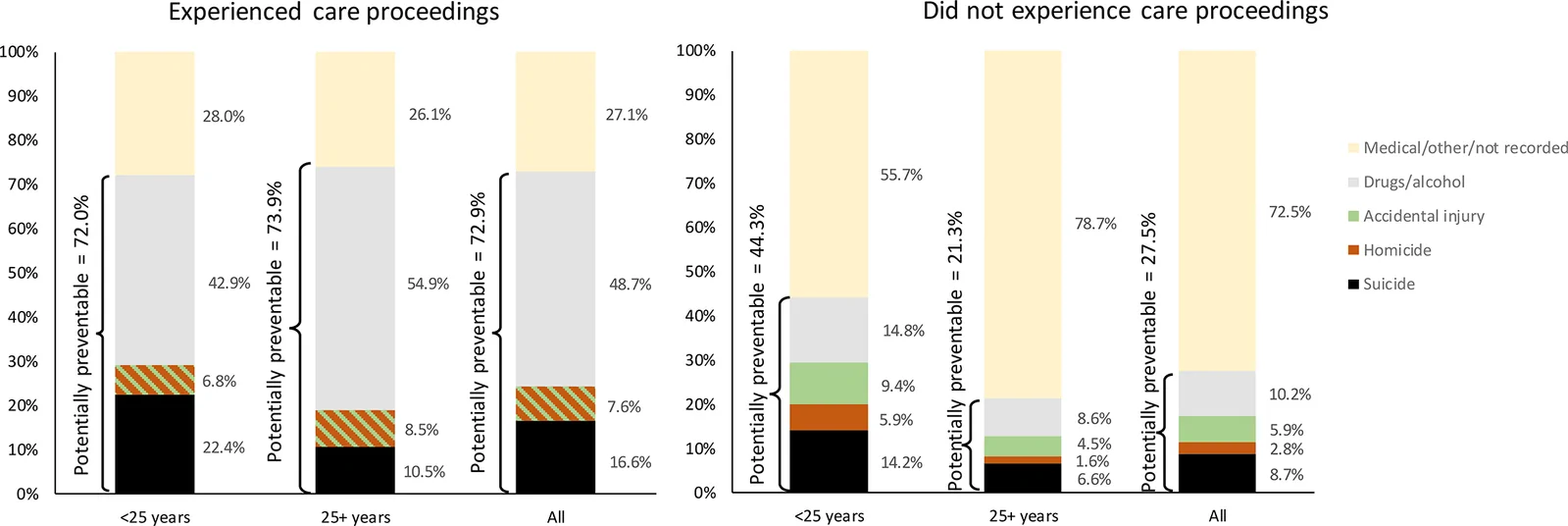
Percentage of deaths related to potentially preventable causes (suicide, homicide, drugs/alcohol, accidental injury) or medical, other or unknown causes by experience of care proceedings and maternal age at first live birth. Deaths due to accidental injury and homicide among mothers who experienced care proceedings are merged due to low cell counts of both. Underlying frequencies are available in online supplemental table 1.

### Objective 3: mortality following first care proceedings

Kaplan-Meier estimates of cumulative mortality in the 10 years following proceedings are given in figure 2 and online supplemental table 2. The overall cumulative incidence was 2.1%. Stratified analyses by various factors highlighted that there were higher cumulative mortality rates with: increasing age at the start of proceedings; pre-delivery health conditions (with the exception of intellectual disability) and adversity-related admissions; and the final legal order at the end of proceedings. In proceedings where the case was discharged, no order was made, or a family assessment order or supervision order was made (ie, where the child likely remained at home), the cumulative incidence was around 0.8% to 0.9%. This compares to between 2.4% and 2.6% where orders were made implying child removal.

**Figure 2:**
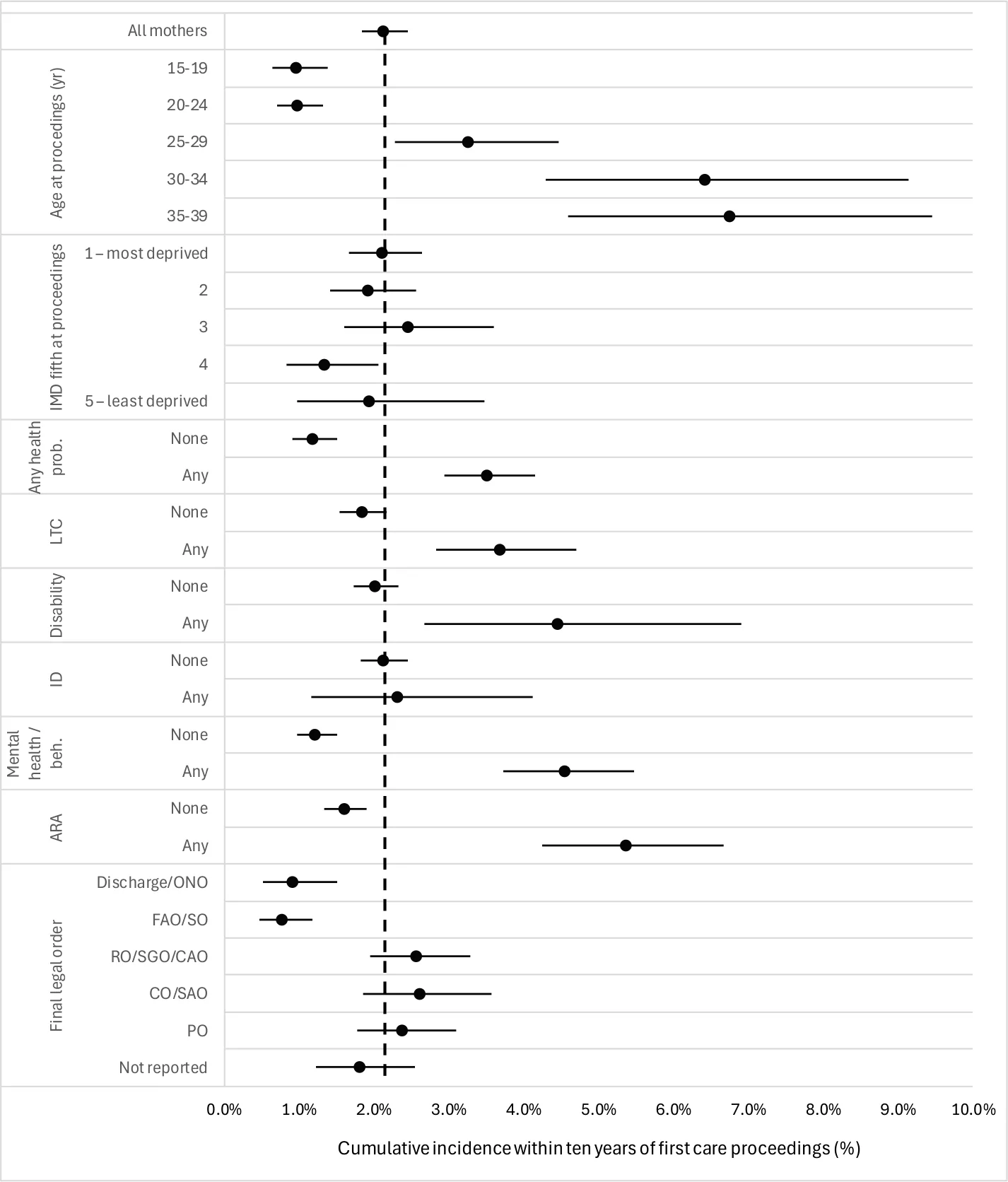
Unadjusted cumulative incidence of mothers’ mortality within 10 years of first care proceedings, by age, deprivation, health problems recorded in the 3 years before care proceedings, and final legal order at end of care proceedings. ARA adversity-related admission; beh. behavioural; CAO child arrangements order; CO care order; FAO family assessment order; ID intellectual disability; IMD Index of Multiple Deprivation; LTC long-term condition; ONO order of no order; PO placement order; prob. Problem; RO residence order; SAO secure accommodation order; SGO special guardianship order; SO supervision order. Underlying frequencies are available in online supplemental table 2.

## Discussion

We found a starkly higher risk of premature death among mothers who experienced care proceedings compared with those who did not. Once age-standardised, and accounting for immortal time bias, women died at a rate 21 times higher in periods following care proceedings compared with women without proceedings. Most deaths among the care proceedings group were potentially preventable, being related to suicide, homicide, drugs/alcohol, or accidental injury in 73% of cases, regardless of age. By contrast, among women who had not experienced care proceedings, the percentage of deaths related to these causes was 21% for mothers aged 25 years or older at their first live birth and 44% for mothers aged under 25. The substantially higher percentage of deaths related to these adversity causes and the lack of an association with age further underscore the extreme vulnerability of mothers who experience care proceedings. Our findings suggest that action could be taken to prevent most of these deaths. That we observed higher mortality rates with increasing age among mothers with proceedings but not among those without further underscores the vulnerability of this group.

As noted in the introduction, mechanisms explaining a higher risk of death are complex, including pre-existing poorer mental and physical health, a theory supported by our data. It may also be that child removal increases maternal mortality risk.^5–7^ The profound impact of child removal has been described, including long-lasting grief, guilt and stigma; loss of identity as a parent; a sense of failure; and adverse health consequences—all without access to post-removal support.^10^ Quantitative evidence further indicates outcomes for mothers experiencing custody loss may be worse than those who experience child death.^6 8 21^ We found that in care proceedings, where orders were made related to child removal, the 10-year mortality risk was between 2.4% and 2.6%, compared with 0.8% to 0.9% where orders were made where the child likely remained at home. The data are therefore consistent with an increased risk of maternal death due to child removal, though such interpretation must be extremely cautious as other contributing factors are likely driving ‘selection’ into these orders, such as more severe previous abuse and health problems. In other words, it is impossible using these data to estimate the causal effect of court decision on maternal mortality. Additionally, no data were available on actual placements (for example, a care order can include a placement at home and between 6% and 9% of children looked after are placed with parents depending on calendar year).^22 23^ This could be remedied with linkage to children’s social care data,^24^ provided these can be improved by inclusion of natural identifiers enabling linkage of younger children’s data.

A summary of previous research on mortality risk among similarly deprived mothers can be found in table 3.^4–7 25^ Our crude mortality rate (2.3/1000 person-years) among mothers experiencing care proceedings was slightly lower than the rates of 3.7/1000 person-years and 3.8/1000 person-years observed in Manitoba^6^ and Sweden,^7^ respectively, for mothers with children removed to care (including via non-judicial routes). Our estimated 10-year mortality risk (2.1%) was lower than those of Pearson *et al*^4^ (among mothers accessing south London mental health services with care proceedings, 3.1%) and Guttmann *et al*^25^ (among mothers of infants with neonatal abstinence syndrome, 5.1% in England and 4.6% in Ontario, Canada). However, the relative difference (age-standardised mortality ratio of 21.0) was higher in our study than in others (HRs of 2.2 to 12.1). In these other studies, comparison groups were similarly disadvantaged, underestimating relative mortality differences vis-à-vis the general population. For example, Pearson *et al*^4^ compared with other mothers accessing secondary mental health services, and Wall-Weiler *et al*’s^5 6^ comparison groups in Manitoba consisted of biological sisters who did not have children removed or mothers receiving other forms of social care intervention. Additionally, some studies employed matching and adjusted for covariates which likely attenuated differences. We provide unadjusted results, meaning our findings reflect actual real-world risks.^13^

**Table 3:** Summary of previous studies examining maternal mortality following loss of child custody or in similarly deprived populations

Study	Jurisdiction	Cohort and data sources	Exposure group	Comparison group(s)	Findings (rates)	Findings (relative difference)
Pearson *et al* (2022) (all-cause mortality: cohort study of women using mental health services)^4^	South London, England	Women using mental health services in four south London boroughs, 2005 to 2020: South London and Maudsley Biomedical Research Centre Case Register linked to Cafcass data	Mothers with care proceedings between 2007 and 2019 and accessing mental health services	Women aged 16–55 years accessing mental health services, matched on type of service accessed and calendar year	Estimated 10 year mortality 3.12% (95% CI 2.47% to 3.94%) among mothers with proceedings and 1.46% (95% CI 1.27% to 1.68%) among women without	HR of death 2.15 (95% CI 1.68 to 2.74) accounting for immortal time bias and adjusting for age (mothers with proceedings compared with women without)
Guttmann *et al* (2019) (all-cause and cause-specific mortality: whole population cohort)^25^	England and Ontario, Canada	Whole population cohort of women aged 12 to 49 with a live birth between 2002 and 2012: linked national hospital administrative data and death registrations	Mothers who gave birth to a child with recorded neonatal abstinence syndrome	Mothers who gave birth to a child without recorded neonatal abstinence syndrome	Estimated crude 10-year mortality among exposure group: 5.1% (95% CI 4.7% to 5.6%) in England and 4.6% (95% CI 3.8% to 5.5%) in Ontario. Among controls: 0.42% (95% CI 0.41% to 0.42%) in England and 0.40% (95% CI 0.38% to 0.41%) in Ontario	Crude HR for all-cause mortality: 12.1 (95% CI 11.1 to 13.2) in England and 11.4 (95% CI 9.7 to 13.4) in Ontario; age adjustment did not reduce the HRs.
Wall-Wieler *et al* (2018) (suicide: discordant sibling analysis)^5^	Manitoba, Canada	Women whose first child was born in Manitoba between 1992 and 2015: linked population administrative data including child and family services and mortality data	Mothers with children removed to care by child protective services (which may include non-court-based routes)	(1) A sister with the same biological mother without a child removed to care; (2) mothers without a sister in the cohort who received support from child protective services but did not have a child removed	Suicide attempt rate per 1000 person-years among those with a child taken into care: 47 (95% CI 37 to 49*); among sisters: 18 (95% CI 13 to 27). Suicide completion rate per 1000 person-years among those with a child taken into care: 9.5 (95% CI 5.8 to 15.6); among sisters: 1.9 (95% CI 0.6 to 5.9)	Children taken into care vs sisters (incidence rate ratios): suicide attempt: crude: 2.55 (95% CI 1.68 to 3.86); adjusted: 2.15 (95% CI 1.40 to 3.30). Suicide completion: crude: 4.99 (95% CI 1.55 to 16.02); adjusted: 4.46 (95% CI 1.39 to 14.33). Adjusted for age at index date, rurality, suicide attempts in 2 years before index date, number of mood/anxiety disorder diagnoses; number of substance use disorder diagnoses. Similar results when comparing mothers with children removed to care with mothers who otherwise received support from child protective services but without children removed to care
Wall-Wieler *et al* (2018) (all-cause mortality: discordant sibling analysis)^6^	Manitoba, Canada	Women whose first child was born in Manitoba between 1992 and 2015: linked population administrative data including child and family services and mortality data	Mothers with children removed to care by child protective services (which may include non-court-based routes)	(1) A sister with the same biological mother without a child removed to care; (2) mothers who experienced death of a child	All-cause mortality rate per 1000 person-years among those with a child taken into care: 3.7 (95% CI 2.9 to 4.7); among controls: 1.3 (95% CI 0.9 to 2.0)	In fully adjusted models, the HR of death for all causes comparing women with a child removed to biological siblings without was 3.23 (95% CI 1.62 to 6.41). Avoidable mortality: 3.46 (95% CI 1.41 to 8.48). Unavoidable mortality: 2.92 (95% CI 1.01 to 8.44). Models were adjusted using a family fixed effect and using high-dimensional propensity scores to control for sociodemographic and health factors in the 2 years before index date (whether mother had moved, welfare receipt, deprivation, rural/urban, plus over 500 health covariates selected statistically)
Wall-Wieler *et al* (2018) (all-cause mortality: Sweden)^7^	Sweden	Mothers and fathers whose oldest child was born in Sweden between 1990 and 2012: linked population registries	Biological parents who had a child placed into care (which may include non-court-based routes) before 2012	Biological parents who did not have a child placed into care before 2012, matched on parental year of birth, child’s year of birth and child’s birth order	Among mothers: all-cause mortality rate per 1000 person-years among those with a child taken into care: 3.8 (95% CI 3.4 to 4.2); among controls: 0.6 (95% CI 0.6 to 7.0). Among fathers: all-cause mortality rate per 1000 person-years among those with a child taken into care: 5.7 (95% CI 5.3 to 6.2); among controls: 1.4 (95% CI 1.3 to 1.5)	Among mothers: adjusted HRs, comparing those with a child taken into care with those without a child taken into care: all-cause mortality: 3.25 (95% CI 2.54 to 4.16); avoidable mortality: 3.18 (95% CI 2.36 to 4.27). Among fathers: Adjusted HRs, comparing those with a child taken into care with those without a child taken into care: all-cause mortality: 1.41 (95% CI 1.20 to 1.66); avoidable mortality: 1.59 (95% CI 1.31 to 1.94)

* A confidence interval of 38 to 39 is reported, though the upper band is presumably a typographical error.

Cafcass, Children and Family Court Advisory and Support Service .

The strengths of this study include the use of whole population data, providing a cohort of 2.7 million first-time mothers all with at least 3 years’ follow-up available for analysis across healthcare, family court and mortality data. Mothers with deliveries in hospital data are representative of all women delivering in England, but the methodology excludes mothers who delivered their first child outside of the NHS, either within private hospitals (<1% of all births), at home (~2% of all births) or outside of England. Our study is also at risk of left-censoring in that births before 1 April 1997 could not be identified, affecting a minority of mothers in earlier years of data.^14^ We could also not observe emigration. Long-term physical or mental health problems are not fully ascertained in hospital data and we likely underestimated the extent to which mothers experienced drug/alcohol use and mental health conditions. To improve case-ascertainment for these conditions, linkages to other datasets are required, including primary care and mental health service data.

We were unable to examine mothers who had children looked after under so-called voluntary arrangements with children’s social care, or mothers who experienced custody loss via private family court proceedings. These mothers were counted among the control group in our study. Assuming that these mothers, too, have a higher mortality risk given they have been shown to have other poorer health outcomes,^12 26^ this would serve to underestimate the relative risk of death in mothers experiencing child removal compared with controls.

Preventive interventions might reduce the need for care proceedings. General practitioners and health visitors are the first point of contact for mothers at their first pregnancy and provide the bedrock of universal healthcare for children and families. These universal healthcare services underpin targeted programmes such as health visiting and the Family Nurse Partnership for first-time teenage mothers.^27^ Family support centres, such as Sure Start centres or family hubs,^28^ may also offer mental health, domestic abuse and substance use support, parenting training, or advice about housing and financial problems. However, both universal and targeted services suffer from rising demands, understaffing, and patchy commissioning. For example, a 2022 study reported Family Nurse Partnership commissioning in only two-thirds of local authorities in England, with capacity to enrol only one-quarter of all first-time teenage mothers intended for support.^27^ A recent confidential inquiry found individual and systemic barriers to accessing holistic, trauma-informed care among mothers with social care involvement who died in the first year following childbirth.^12^ These barriers included problems with: risk assessment only focusing on the child; tick-box style medical risk assessments to the detriment of biopsychosocial approaches; timely access to medication management; uncoordinated healthcare and limited communication across agencies; and lack of safeguarding competency among healthcare staff. Further, intensive support primarily focuses in the first 1000 days whereas our findings indicate intensive support may be needed beyond this window, and providing support may be difficult where mothers lack trust in public services and/or are stigmatised.^10^ Ongoing monitoring and evaluation of mortality and other health outcomes could be supported by routine anonymised linkage between Cafcass and hospital data.

Findings also have implications for family law and policy, including the need to adopt a holistic approach to the family and their problems. The Family Drug and Alcohol Court, which provides a ‘problem-solving’ approach to adjudication in contrast to the family court’s traditional adversarial nature, have shown promise in addressing the underlying problems that lead to proceedings in the first place with improved drug use outcomes among parents and higher family reunification rates.^29^ However, there are only 13 such courts operating in 35 local authorities, which require specialist training and provision. A holistic view should also consider private proceedings (usually between parents following relationship breakdown), which our study did not examine. Those involved in private law proceedings also have poorer mental and physical health and experience deterioration in health as a result of proceedings.^30^ This is especially so in cases involving domestic abuse, estimated to be the majority of private proceedings.^30^ Research is needed on mothers involved in both public and/or private proceedings. Such research could also make use of mother–baby links in hospital data^31^ to examine outcomes for children, other datasets including mental health data in England and substance misuse and primary care data in Wales, and data on fathers, who have been understudied to date. Ultimately, a public health approach to family justice is needed that examines why families come to court in the first place, their journeys through the courts and their long-term outcomes following proceedings.^32^

## Supplementary material

10.1136/jech-2026-225930 online supplemental table 1

## Data Availability

Data may be obtained from a third party and are not publicly available.
