# Supplementary material for: 10-year mortality among first-time mothers involved in family court care proceedings in England: cohort study using linked administrative hospital, mortality and family court records
Source: J Epidemiol Community Health. 2026 Jun 23;80(11):e225930. doi: 10.1136/jech-2026-225930 (PMC13691665; doi:10.1136/jech-2026-225930)
Supplement: Supplementary file 1 [file jech-80-11-s001.pdf]

Supplementary Tables

Supplementary Table 1a. Number and percentage of deaths by type of death for mothers who experienced care proceedings by age at first live birth (underlying data for Figure 1)

	Experienced care proceedings		
	**<25 years**	**25+ years**	**All**
Potentially preventable	116 (72·0%)	113 (73·9%)	229 (72·9%)
Suicide	36 (22·4%)	16 (10·5%)	52 (16·6%)
Homicide / accidental injury	11 (6·8)	13 (8·5%)	24 (7·6%)
Drugs/alcohol	69 (42·9%)	84 (54·9%)	153 (48·7%)
Medical/other/not recorded	45 (28·0%)	40 (26·1%)	85 (27·1%)

Deaths due to accidental injury and homicide among mothers who experienced care proceedings are merged due to low cell counts of both.

Supplementary Table 1b. Number and percentage of deaths by type of death for mothers who did not experience care proceedings by age at first live birth (underlying data for Figure 1)

	Did not experience care proceedings		
	**<25 years**	**25+ years**	**All**
Potentially preventable	612 (44·3%)	793 (21·3%)	1,405 (27·5%)
Suicide	197 (14·2%)	245 (6·6%)	442 (8·7%)
Homicide	81 (5·9%)	60 (1·6%)	141 (2·8%)
Drugs/alcohol	204 (14·8%)	319 (8·6%)	523 (10·2%)
Accidental injury	130 (9·4%)	169 (4·5%)	299 (5·9%)
Medical/other/not recorded	771 (55·7%)	2,927 (78·7%)	3,698 (72·5%)

Supplementary Table 2. Number of deaths within ten years of start of care proceedings and unadjusted cumulative incidence of mothers' mortality within ten years of first care proceedings, by age, deprivation, health problems recorded in the three years prior to care proceedings, and final legal order at end of care proceedings (underlying data for Figure 2)

		Deaths	10-year mortality estimate, % (95% confidence interval)
Total		314	2.13 (1.84, 2.45)
Age at delivery (years)	15-19	34	0.96 (0.65, 1.38)
	20-24	51	0.98 (0.71, 1.32)
	25-29	72	3.25 (2.29, 4.48)
	30-34	58	6.43 (4.29, 9.15)
	35-39	58	6.76 (4.60, 9.46)
IMD fifth	1 – most deprived	129	2.11 (1.66, 2.64)
	2	63	1.93 (1.42, 2.56)
	3	40	2.45 (1.60, 3.60)
	4	21	1.34 (0.83, 2.07)
	5 – least deprived	12	1.94 (0.98, 3.48)
Health problems in 3 years prior to case start	None	101	1.18 (0.91, 1.50)
	Any[a]	213	3.51 (2.94, 4.16)
Long-term	None	218	1.84 (1.54, 2.17)
	Any	96	3.69 (2.83, 4.70)
Disability	None	283	2.01 (1.73, 2.32)
	Any	31	4.46 (2.67, 6.91)
Intellectual disability	None	301	2.13 (1.83, 2.45)
	Any	13	2.31 (1.16, 4.13)
Mental health/ behavioural	None	128	1.22 (0.97, 1.50)
	Any	186	4.55 (3.73, 5.48)
ARA (Any)	None	204	1.60 (1.34, 1.90)
	Any	110	5.37 (4.25, 6.68)
Final legal order	Discharge/ONO	14	0.91 (0.51, 1.51)
	FAO/SO	19	0.76 (0.47, 1.19)
	RO/SGO/CAO	102	2.56 (1.96, 3.29)
	CO/SAO	68	2.61 (1.86, 3.58)
	PO	78	2.37 (1.78, 3.09)
	Not reported	33	1.80 (1.23, 2.56)

[a] Any health problem includes long-term conditions, disability, intellectual disability, mental health/behavioural and any ARA. All are potentially overlapping. ARA adversity-related admission; CAO child arrangements order; CO care order; FAO family assessment order; IMD Index of Multiple Deprivation; ONO order of no order; PO placement order; RO residence order; SAO secure accommodation order; SGO special guardianship order; SO supervision order.
